# Improved donor chimerism in relapse myelofibrosis post allogenic stem cell transplant with azacitidine and oral decitabine—First case report

**DOI:** 10.1002/jha2.611

**Published:** 2022-11-16

**Authors:** Verna Cheung, Fotios V. Michelis, Hassan Sibai

**Affiliations:** ^1^ Princess Margaret Cancer Centre Toronto Ontario Canada; ^2^ University of Toronto Toronto Ontario Canada; ^3^ Hans Messner Allogeneic Transplant Program Princess Margaret Cancer Centre, University Health Network Toronto ON Canada

**Keywords:** allogenic stem cell transplant, hypomethylating agent, myeloid lineage chimerism, primary myelofibrosis

## Abstract

To date, allogenic stem cell transplant (ASCT) remains the only potential curative option for patients with primary myelofibrosis (PMF). However, relapse rates and associated mortality remain a concern. A second ASCT may not be feasible due to advancing age, declined functional status, donor unavailability, toxicities associated with a second ASCT. Herein, we report the first case of utilizing initially azacitidine and subsequently oral decitabine + cedazuridine (decitabine), in the context of relapsed PMF post‐ASCT. Utilizing both hypomethylating agents provided disease control and improved donor/myeloid lineage chimerism levels, and the patient also remained transfusion independent, with preserved functional status and quality of life.

1

Primary myelofibrosis (PMF) is a myeloproliferative neoplasm (MPN) driven by clonal disorders of hematopoietic progenitor cells with a risk of transformation to acute myeloid leukemia (AML) [1]. Despite advances in allogenic stem cell transplant (ASCT), relapse following ASCT for MF remains concerning [1, 2]. The type of donor such as a haploidentical‐donor can have an impact on outcomes such as non‐relapse mortality and graft failure [3, 4]. However, once disease relapse (DR) occurs post‐ASCT, treatment options are limited to donor lymphocyte infusion (DLI) and second ASCT; and overall survival (OS) is poor [1, 2, 3, 4].

Studies have demonstrated an association between decrease donor chimerism (DC)/myeloid lineage chimerism (MLC) and relapse [3, 5, 6, 7]. Studies also show hypomethylating agents (HAs), that is, azacitidine and decitabine, are effective as salvage therapy (ST) in the setting of post‐ASCT relapse by inducing immune response and improving MLC levels [8, 9, 10, 11, 12, 13, 14, 15].

Here we report, to our knowledge, the first case of a patient with high‐risk PMF relapsed post‐ASCT using a haploidentical‐donor, who achieved disease control with improved MLC, using initially azacitidine and subsequently oral decitabine + cedazuridine (decitabine), remains alive after 30 months since DR.

Patient has a 72F JAK2+ PMF relapsed post‐ASCT after 3 years. On initial presentation, she was 66, JAK2+ PMF with anemia, blast, and splenomegaly. In September 2017, she underwent ASCT with a haploidentical‐donor. On day 60 post‐ASCT, DC was 100%. The marrow reported cellularity 60% with MF2–3 of 3, JAK2 undetectable.

In April 2020, DC dropped to 79.6% in bone marrow biopsy (BMB). In May 2020, it revealed hypercellular marrow with MF2, with blast 7%, with JAK2+, indicating DR with progression. A second transplant was considered highly risky given her advanced age and no alternative donor. DLI was also considered highly risky. Therefore, azacitidine was initiated to provide disease control [8, 9].

After three cycles of azacitidine, BMB showed normocellular marrow with MF1–2, with <5% blast and T‐cell lineage chimerism (TLC) at 100% and MLC at 89.3%. After seven cycles of azacitidine, the patient began to experience leukopenia and neutropenia, with TLC at 100%, but MLC declined to 76%. Repeat BMB showed a chronic phase of MF with no blast. But given persistent neutropenia azacitidine was held.

By September 2021, BMB demonstrated PMF with a blast nearing 10%, indicating MPN accelerated‐phase. TLC was 99.1%, MLC dropped to 2.8%. Therefore, decitabine was initiated as a second line HA. After four cycles of decitabine, BMB showed chronic phase PMF with MF3, and blast 1%. Unfractionated chimerism was 44.8%. The patient remained well, transfusion independent, and cycle 5 restarted after a delay of 12 weeks due to cytopenia, with a reduced dose (Table 1 and Graph 1).

**TABLE 1:** Timeline of bone marrow biopsies, and chimerism levels

**Year**	**2016—Initial diagnosis**							**Aug 2017 pretransplant marrow**							**Nov 2017—60‐ day post ASCT marrow**							**May 2020—pre‐ azacitidine treatment marrow**							**April 2021— persistent cytopenia post seven cycles of azacitidine**							**Dec 2021— repeat marrow after 1 cycles of oral decitabine**						
Labs	Hb	hct	MCV	PLT	Wbc	Neut	blast	Hb	hct	MCV	PLT	WBC	Neut	Blast	Hb	Hct	MCV	PLT	WBC	Neut	blast	Hb	hct	mcv	Plt	Wbc	neut	blast	Hb	hct	mcv	Plt	Wbc	Neut	Blast	Hb	hct	Mcv	Plt	WBC	Neut	Blast
	86	0.26	84.7	280	9.6	7.6	0	68	0.21	88	114	2.9	1.5	1%	92	0.26	88.1	95	5.7	3.9	1%	103	0.32	90.7	116	1.5	0.7	3%	98	0.31	93	193	1.3	0.6	1%	71	0.21	93	128	0.5	0.2	0
Bone marrow biopsy	95% Cellularity, erythropoiesis hypercellular. Granulopoiesis hypercellular, with left shift. Megakaryopoiesis hypercellular, abnormal distribution with tight clusters. Megakaryocytes show cloud‐like nuclei. MF2 by reticulin and trichrome staining							95% Cellularity, erythropoiesis hypocellular, granulopoiesis hypercellular, megakaryopoiesis normocellular, atypical hyperchromatic chromatin, in tight clusters. MF3 by reticulin and trichrome staining							Approximately 60% cellularity, erythropoiesis shows some area of left shift, granulopoiesis, prominent eosinophils, megakaryopoiesis appears normal. MF2 out of 3 by reticulin and trichrome staining							40% Cellularity, moderately hypercellular, erythropoiesis normocellular, granulopoiesis normocellular, megakaryopoiesis relatively hypercellular, a moderate number of atypical forms, including large cells with nuclear hypolobation and loose clustering. CD34+ blast cells 7%. MF2–3							Hypercellular marrow (80%), erythropoiesis hypercellular, granulopoiesis hypocellular, megakaryopoiesis relatively hypercellular, with tight clustering and atypia. MF grade 1–2. Less than 5% blast							Hypercellular marrow with megakaryocytes are large, hypersegmented/metachromatic, in occasional clusters. MF grade 2, CD34+ blast 5%						
Molecular	JAK2 TET2							JAK2 TET2							JAK2 not detectable							JAK2, TET2 and BCOR							Not repeated							Not repeated						
Cytogenetics	N/A							N/A							N/A							N/A							N/A							N/A						
Chimerism	N/A							N/A							Complete engraftment—100,2%							Post‐transplant CD3 (T‐cell lineage) chimerism 100% Post‐transplant CD66b/CD33 (myeloid lineage) chimerism—51.9%							Post‐transplant CD3 (T‐cell lineage) chimerism 100% Post‐transplant CD66b/CD33 (myeloid lineage) 66.2%							Post‐transplant CD3 (T‐cell lineage) chimerism 99.2% Post‐transplant CD66b/CD33 (myeloid lineage) chimerism 2.8%						
Follow‐up bone marrow biopsy	N/A							N/A							**Reason: April 2020 follow‐up marrow for cytopenia**							**Reason: Oct 2020 reassess treatment response**							**Reason: September 2021 repeat marrow due to persistent anemia and leukopenia**							**Reason: May 2022 repeat marrow to assess treatment response due to prolonged anemia and leukopenia**						
															Cellularity 50%, erythropoiesis relatively hypercellular, granulopoiesis relatively hypocellular, megakaryopoiesis normocellular, rare loose clusters, with cloud‐like nuclei. MF0 by trichrome and reticulin staining Disease relapsed based on decreased chimerism, and presence of JAK2+							Normocellular marrow (70%), erythropoiesis normocellular, granulopoiesis normocellular, megakaryopoiesis relatively hypercellular, MF0 with focal MF1–2							Hypercellular marrow (90%), erythropoiesis relatively hypercellular, granulopoiesis hypocellular, megakaryopoiesis, morphology—occasional large megakaryocytes with complex and bulbous nuclei. MF2–3+ out of 3. Blast from 5 to upward of 10%							Hypercellular marrow 100% cellularity, erythropoiesis normocellular, granulopoiesis hypercellular, megakaryopoiesis hypercellular, megakaryocytes appear in clusters and as single cells, are large, have atypically lobulated nuclei with increased nuclear cytoplasmic index. MF3/3 CD34+ blast 1%						
															Chimerism: 79.6%							Post‐transplant CD3 (T‐cell lineage) chimerism 100% Post‐transplant CD66b/CD33 (myeloid linage) chimerism 89.3%							Post‐transplant CD3 (T‐cell lineage) chimerism 99.1% Post‐transplant CD66b/CD33 (myeloid linage) chimerism 2.2%							Post‐transplant unfractionated chimerism 44.8%						
															Molecular: JAK2+							Molecular not repeated							Molecular not repeated							Molecular not repeated						
															hb	hct	mcv	plt	wbc	neut	blast	hb	hct	mcv	plt	wbc	neut	blast	Hb	Hct	Mcv	Plt	Wbc	Neut	Blast	Hb	hct	MCV	Plt	Wbc	neut	Blast
															103	0.32	87.5	104	1.8	0.6	0	102	0.33	95.1	171	2.9	1.0	1%	87	0.27	96.9	229	0.8	0.4	1%	89	0.29	103	252	0.9	0.3	2%

Abbreviation: ASCT, allogenic stem cell transplant.

**GRAPH 1:**
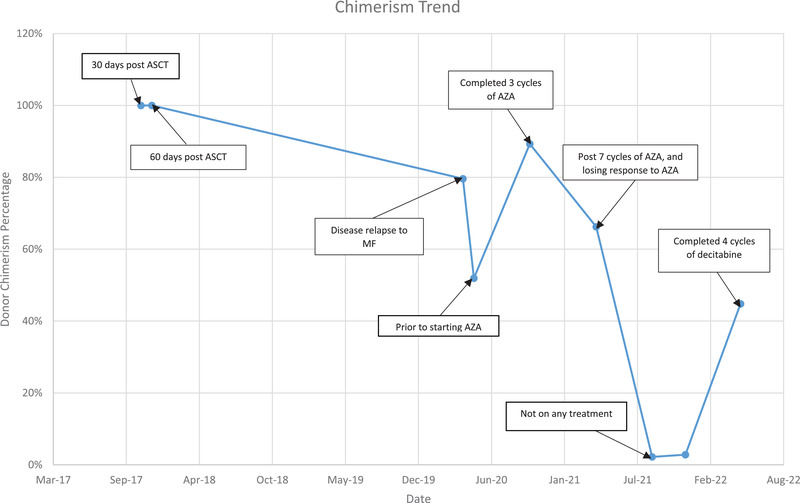
Donor chimerism levels and treatment with hypomethylating agents

Despite advances in novel treatments for MPNs, ASCT remains the only curative option; however, retrospective studies have suggested relapse rate range from 18.3% to 25% at 5‐year post‐ASCT, with morality related to DR up to 41% [2, 15]. A second transplant may not be possible for multiple reasons, such as increased toxicities, patient advanced age, and declined performance status [2, 9, 10, 11, 12, 13, 14, 15]. Monitoring chimerism can be helpful to detect early relapse and for early intervention with ST, as decreased MLC is associated with DR, decreased progression‐free‐survival, and OS [2, 5, 6, 7, 12]. Several studies have shown that ST with HAs of either azacitidine or decitabine improves MLC, which is associated with improved OS [9, 10, 11, 12, 13, 14, 15].

Azacitidine has been shown to provide improved DC [7, 8] and an appropriate ST for patients with DR post‐ASCT [9, 10, 11, 12]. Sumi et al. demonstrated that after three cycles of azacitidine, a patient with MF relapsed 9‐year post‐ASCT achieved partial cytogenetic response with improved DC levels [9]. Another study utilizing azacitidine as ST for patients with various myeloid malignancies with DR post‐ASCT demonstrated a response rate of 30%–33%, and responses in both studies were defined as improved blood counts, whereas disease remained in complete (CR) or partial (PR) remission; the 2‐year OS was 25%–29% [10]. Unfortunately, chimerism levels were not available, and some patients also received DLI as part of ST to achieve CR or PR [10]. Similarly, a single‐center prospective study demonstrated improved relapse‐free‐survival in 28 patients with myeloid malignancies who underwent ASCT [11]. Amongst these 28 patients, 10 had DR post‐ASCT with >30% blast, with a use of azacitidine, 30% achieved CR [11]. Fourteen received azacitidine as a preemptive treatment, either had minimal residual disease or mixed chimerism post‐ASCT. Six of 14 achieved full DC and complete cytogenetic remission, and 1‐year survival for this group was 74%. These findings provide support for the use of HA in early stages of DR [10, 11].

A pilot study administered low‐dose decitabine to 14 patients who have undergone ASCT for either high‐risk MDS or AML [12]. These patients had DC levels from 59% to 97% between day 30 and 180 post‐ASCT [12]. With a 6‐month use of low‐dose decitabine, 10 out of 14 patients achieved full DC with no major complications, thus suggesting that decitabine as monotherapy may be effective in reversing declining chimerism, which helps to prevent DR post‐ASCT [12]. Ganguly et al. also demonstrated that using low‐dose decitabine for eight patients with either MDS or AML with declining DC (<80%), three patients achieved full chimerism, whereas two required DLI in addition to decitabine [13]. In another study of a patient with MDS/MPN overlap syndrome, at day‐90 post‐ASCT, BMB showed DC decreased from 100% to 7%, with 3% blast, indicating DR. After two cycles of decitabine, full DC was achieved [14]. Overall, there is evidence supporting both azacitidine and decitabine as ST post‐ASCT, especially in early morphologic, cytogenetic, or chimeric relapse [9, 12, 13, 15]. However, response rates vary greatly across studies from 14% to 75%, and further investigations are needed [14].

This is the first case demonstrating response and improving MLC to oral decitabine after progression on azacitidine for relapsed PMF post‐ASCT. Although during the time that azacitidine was held due to cytopenia, the patient did progress to accelerated phase MPN with 10% blast, disease control and improved MLC were re‐achieved through decitabine. Furthermore, the patient remained transfusion independent, with preserved performance and functional status. Although our findings are limited to this case report, such positive patient outcomes do warrant the further investigation of the potential use of HAs as ST relapsed post‐ASCT, as it potentially allows for a possible prolongation of survival with preserved quality of life.

## CONFLICTS OF INTEREST

The authors have no conflicts of interest to disclose.

## FUNDING INFORMATION

The authors received no specific funding for this work.

## ETHICS STATEMENT

Consent obtained from patient for this case report.

## PATIENT CONSENT STATEMENT

Consent was obtained from the patient.
